# Multi-player electoral engineering and COVID-19 in the polish presidential elections in 2020

**DOI:** 10.1007/s11299-022-00287-7

**Published:** 2022-03-04

**Authors:** Jarosław Flis, Marek Kaminski

**Affiliations:** 1 The Jagiellonian Center for Quantitative Research in Political Science, Jagiellonian University, ul. Wenecja 2, 31-117 Kraków, Poland; 2 Department of Political Science and Mathematical Behavioral Sciences, University of California, 3151 Social Science Plaza, 92697-5100 Irvine, CA USA

## Abstract

The uniqueness of Poland’s experience with the 2020 coronavirus lays in the interplay of two factors: the decisive governmental response to the pandemic, and the overlap of the pandemic with the country’s presidential election scheduled on May 10, 2020. The government’s fast reaction, combined with the citizens’ discipline, resulted in the suppression of the virus’s spread. The ratings of the current President Duda skyrocketed well above 50% needed for re-election in the first round. However, the expectation was that they would be going down with the pandemic and lockdown fatigue. For almost two months, the government tried to organize the elections under the normal schedule while the opposition tried to block them. Finally, the opposition won, and the elections were rescheduled on June 28, with the President Duda’s ratings substantially lower. Nevertheless, in the runoff on July 11, Duda won. Our conclusion goes against the common opinion that electoral engineering is always one-sided. The reconstruction of the pre-electoral political maneuvers shows that many independent players were simultaneously involved in complex engineering, and that the final outcome was hard to predict until almost the very end.

The uniqueness of Poland’s experience with the 2020 coronavirus lays in the interplay of two factors: the decisive governmental response to the pandemic, and the overlap of the pandemic with the country’s presidential election. As determined by the Polish Constitution, on February 5, 2020, the speaker of the Sejm (the Lower House of Parliament) set the day for the presidential election for May 10. At that time, the common sentiment in Poland considered the coronavirus threat as low.

Nearly a month later, the Prawo i Sprawiedliwość (Law and Justice) government, commonly known by its acronym, PiS, swiftly implemented radical measures against the spread of the virus. On March 2, two days before the first case was diagnosed in the country, the Sejm passed a special law, colloquially named the Coronavirus Act, that introduced the legal tools for fighting the pandemic. Between March 8 and March 11, all mass events were effectively banned, and all educational institutions closed. The nation’s borders were then locked on March 15 when the number of confirmed cases reached 119. The unhappy murmurs coming from Brussels and Berlin about this border closure stopped when Germany soon closed its own borders. On March 20, a *state of epidemic* was declared, and on March 24, a partial lockdown was initiated. On April 1, additional restrictions limited people’s outside movements to basic activities, such as shopping for groceries or medicines, going to medical appointments, attending religious services, walking dogs, or commuting to or from necessary work. Requirements were also imposed on the stores that remained open, which were to provide gloves and hand sanitizers.

The developing *coronasaga* increasingly started to interfere strongly with the prospects of the scheduled May 10, 2020, presidential election. The result was an unusual case of electoral engineering.

## Lockdown blitz

**Fig. 1:**
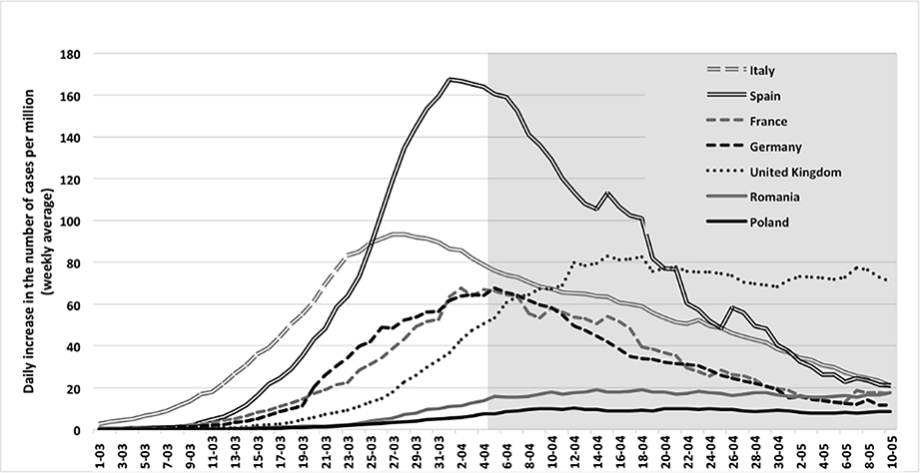
Daily increase in the number of cases (weekly average) per million. (*Note*. The United Kingdom and the six largest EU countries (Italy, France, Germany, Poland, Romania, and Spain) are represented. The grey shading represents the period after April 5. Source: Johns Hopkins CRC (2020); CIA Factbook (2020).)

**Fig. 2:**
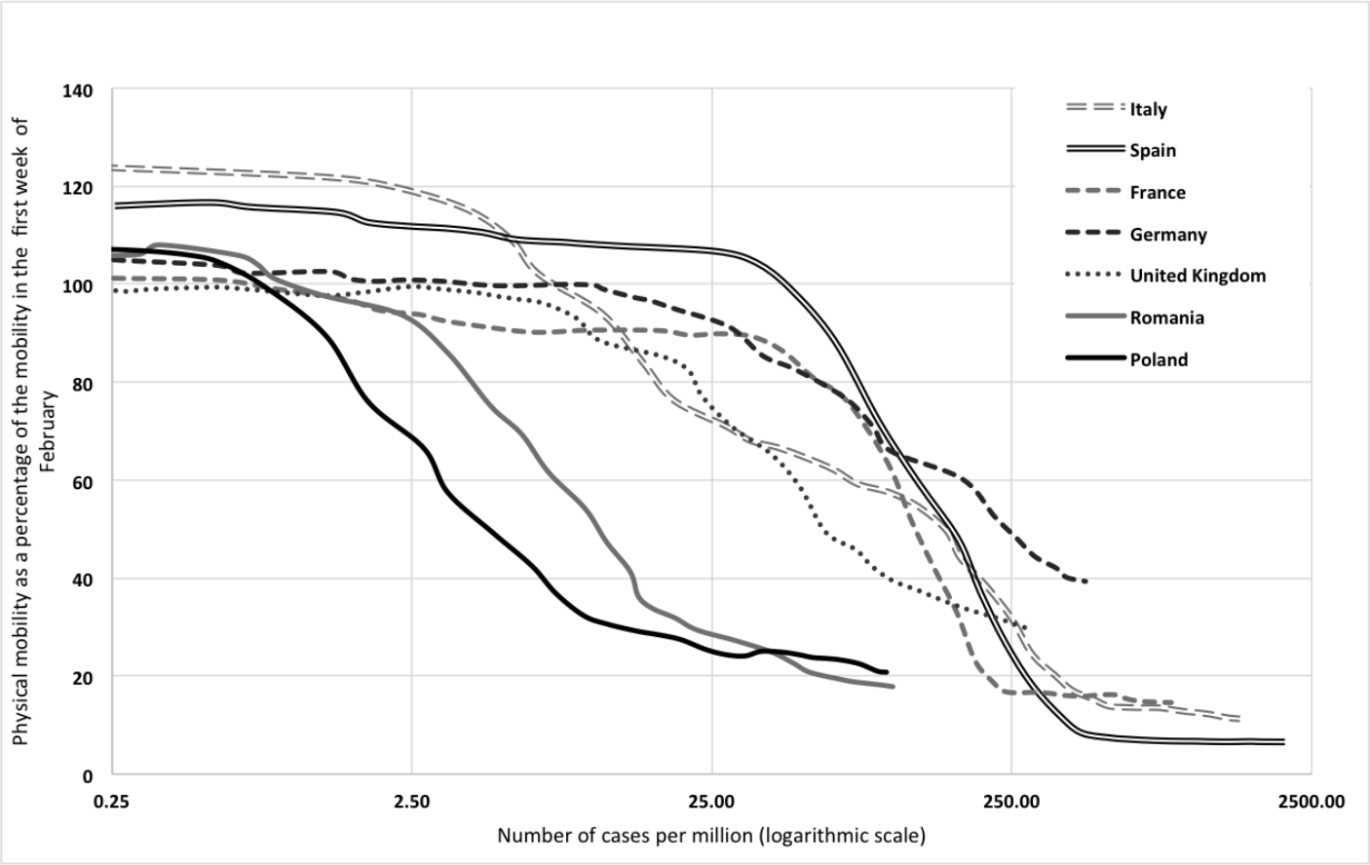
Change in physical mobility by the number of cases per million. (*Note*. “Physical social mobility” is shown as a percentage of such average mobility in the week from January 27 to February 2, 2020. Source: Weekly averages of the variable “walking” found on Apple Mobility Trends from Apple Maps (2020). The graphs end when minimum mobility was reached.)

The government’s fast reaction, combined with the citizens’ discipline, resulted in the suppression of the virus’s spread in Poland. Its containment was especially striking when compared with other large EU countries or the United Kingdom (see Fig. 1). The physical mobility index indicated that Poles quickly limited their activity at low levels of infections, thereby reducing the nation’s infection rate (see Fig. 2). Poland, however, was not the only one; other Central-Eastern European countries, such as Romania, also managed to keep their curves’ flat.^1^

It is possible that Poland’s and other Central-European nations’ determination was strengthened by the grim news coming from Italy and Spain, where the pandemic had developed two to three weeks earlier. French, Germans, and Spaniards waited the longest after the virus took hold before limiting their movements, but then the progression of movement restrictions was rapid. Italians and Britons started quite early limiting their movements but then changed their behavior relatively slowly.

In terms of public opinion, the Polish government’s resolve was rewarded with positive polling: 70% of respondents believed that the authorities had dealt well or quite well with the pandemic versus 6.3% who held the opposite view (United Surveys 2020). Confidence in PiS’s politicians skyrocketed. Łukasz Szumowski, the country’s no-nonsense minister of health, as well as an accomplished cardiologist, led as the country’s most trusted official with a 51.1% rating, versus 15% who said they distrusted him, followed quite closely by President Andrzej Duda and Premier Mateusz Morawiecki (Ibris 2020, April 7). Meanwhile, the various opposition leaders were behind or far behind in their approval ratings. In a representative electoral poll taken at the time, 54.6% of voters declared their support for President Duda against the fragmented opposition candidates’ support, which ranged from 5.5 to 15.5% (Ibris 2020, April 29). With these good numbers, Duda could expect a first-round win in the majority runoff election on May 10.

The economic consequences of the lockdown in Poland were serious but far from disastrous. A consensus among economists was that the nation’s slump would be shallow. A typical article in Bloomberg anticipated a 4.3% fall in Poland’s GDP—the smallest change among all 27 EU economies (Bujnicki and Krajewski 2020). Again, the government’s fast and bold reaction was praised. Additionally, other factors, such as the low price of oil (with Poland as an importer) and the anticipated stay in the country of summer tourists who routinely vacationed in Greece, Turkey, Spain, and Italy, were also expected to help.

That said, the taming of the pandemic—generally interpreted as the ruling government’s spectacular success—created an unexpected electoral dilemma. The ratings of President Duda were near their all-time highs. However, it was also reasonable to expect a future slow slide in the president’s popularity, caused by lockdown fatigue and growing economic distress. Moreover, the slow rise in COVID-19 cases suggested that Poland might need more time than other big EU countries to completely extinguish the pandemic. Any delay in the timing of the election could witness a reversed perception of the country’s comparative success and a dramatic fall in the president’s ratings. Under such circumstances, PiS had strong incentives to keep the original election date, while the opposition demanded a postponement.

## Total electoral engineering

Electoral engineering in Poland was extremely intense in the first decade after the fall of communism in 1989. Virtually all political parties undertook some form of it at one point or another (Kaminski 2002). The classical scenario was that the ruling coalition of parties, or sometimes an ad hoc winning coalition, attempted to engineer the electoral law in their favor while the opposition protested, having limited means to block any undesired changes. However, before the presidential election scheduled in the time of pandemic on May 10, 2020, a different spectacle played out: all political forces would mobilize substantial resources in a game of multilateral electoral engineering.

Historically, Poland’s elections have been organized by the National Electoral Commission and by the National Election Office and its 49 local units. This specialized administration coordinates the activity of close to 2,500 municipalities. Local governments form over 25,000 local electoral commissions, with over 250,000 election volunteers. Initial electoral arrangements for the May 10 elections, including volunteer recruitment, should start in March, and then intensify one month before the election, thus around April 10.

The *state of epidemic* allowed for the introduction of certain restrictions, but the nation’s Constitution did not offer clear recommendations for handling an election during a pandemic. If the situation developed similarly to what occurred in Italy or Spain, then the massive interactions at voting booths could produce a disastrous toll in terms of infections and deaths. Since, at the moment, the infection curve was staying flat, the government tried to run the election as usual, along with certain adjustments. On March 28, the government quickly modified the electoral law by restoring the option of having citizens vote by mail—a law that was introduced in 2014 but then removed in 2017. The opposition criticized the speed of this change and argued that it was inconsistent with an earlier ruling by the Constitutional Tribunal that banned changes to the electoral law less than half a year before an election. There was further criticism that voting by mail was restricted to those who were 60 years or older, a group where the supporters of the ruling party were overrepresented. The opposition considered the election-as-usual approach as unrealistic in any form and demanded postponement. They enlisted the support of the European Union, which quickly started routine anemic reprimanding.

However, while the state of epidemic was introduced only as a regular bill, the Constitution did allow for delaying an election under a *state of natural disaster* or a *state of emergency.* The latter is a more drastic measure than a state of epidemic, while the former is quite similar. Nevertheless, both require moving the election date forward by at least 90 days, something which is not required by a state of epidemic. The legal tools offered by the state of epidemic were introduced during a previous pandemic scare in 2009, when Poland’s present opposition was in power. At the time, present opposition leaders claimed that the tools were a low-cost—in terms of restricting civic liberties—reaction to a pandemic. For the present crisis, the government argued that the state of epidemic was sufficient, while the opposition accused the government’s refusal to declare a state of natural disaster as undemocratic and motivated only by self-interest.

The likely decisive factor that made the PiS government change its plans was the lack of popular support for having the elections proceed as usual. While the government had received high ratings for its coronavirus response, in a March 28 poll, 77.4% of respondents favored postponing the election while only 13.8% were against the idea (Ibris 2020, March 28). All key election decisions were made by April 5. In Fig. 1, the period following April 5 is marked in grey. We could see that the earlier rate of increase was quite steep, and this suggested that around the May 10 election, the epidemic might be at its height, and that the scale of the problems could resemble Italy or Germany.

As mentioned, elections in Poland are organized by local governments, of which about 90% are dominated by opposition or by independent candidates. Expecting dramatic developments, and also convinced that they had strong public support, local governments protested and resisted passively—specifically, by slowing down or stopping their election preparations. There was also a substantial drop in the number of volunteers.

The government responded to this passive resistance by attempting an organizational bypass of the local governments. On April 6, PiS’s MPs presented an amendment to the electoral law that introduced a mail-only elections, with the Polish Post charged with the logistics of running them instead of local governments. The bill was accepted by the Sejm that same day. In the amendment, the number of electoral committees was limited tenfold, and the central administration was designated as the one to oversee the local administration of the elections.

Quite unsurprisingly, the opposition objected and then counterattacked. Various political forces, both at home and abroad, heavily criticized the last-minute changes to the electoral law, including the Supreme Court. Some political analysts argued that the elections were logistically impossible to organize due to Polish Post’s inexperience, and they also said that having the Post run it would result in a spike in the infection rate (Flis and Marcinkiewicz 2020). The government’s counterargument was that the Polish Post handled twice as many pieces of mail in a single week as would be required for a mail-only election. Moreover, while some states, provinces, and countries around the world chose to postpone their elections, others went ahead and held them amid the pandemic. Thus, for example, while Bavaria chose to vote exclusively by mail in the runoff to local elections on March 29, South Korea and the US states of Wisconsin, Arizona, Florida, and Illinois held ordinary primaries or elections.

**Fig. 3:**
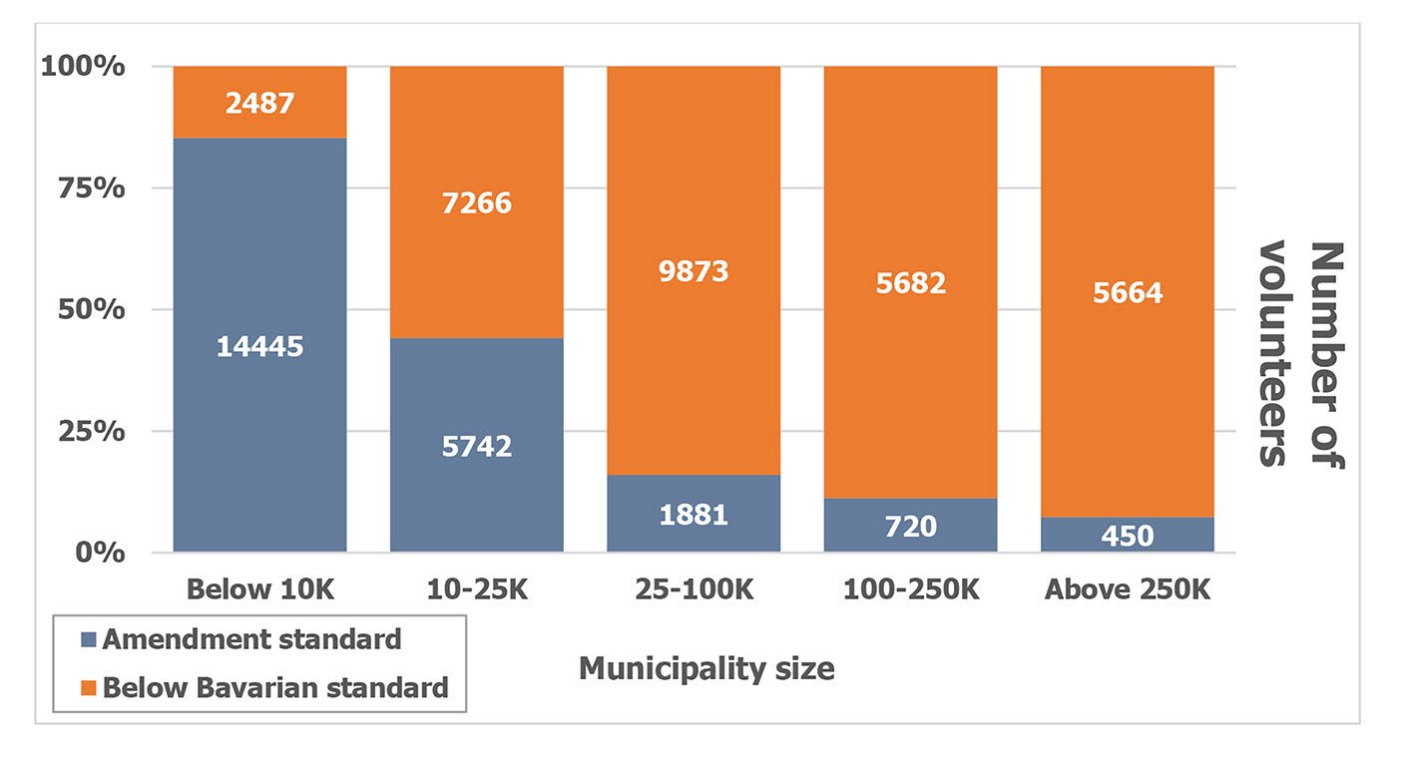
Number of volunteers established by the amendment to the Polish electoral law versus the numbers of actual volunteers for the Bavarian mail-only election by municipality size

Election opponents argued that the logistics needed would lead to mistakes and that the low number of volunteers, as detailed in the electoral law amendment, would slow down the pace of counting votes and would threaten a possible runoff planned two weeks later. The expected deficits of volunteers would be largest in the nation’s biggest cities. Figure 3 shows the number of volunteers in the amendment compared to the number of volunteers for the Bavarian mail-only election by municipality size (Flis and Ciszewski 2020).

The opposition backed up its verbal arguments by creating obstructions. The government’s amendment bill went to the opposition-controlled Senate, where it languished for the maximum-allowed 30 days before finally being rejected. At the same time, the Senate accepted one of the separately passed special bills that canceled the traditional election and that was passed by the Sejm after the amendment. Thus, the old administrative structures were legally released from preparing for the election while the new ones had no legal basis to start working on the election in the new format. A conciliatory proposal was put forward by Deputy Premier Jarosław Gowin, which combined the election delay with extending the president’s term by two years and limiting the number of terms to one. This offer was rejected by the opposition.

Just before the elections date, tensions increased, especially within the ruling coalition. Deputy Prime Minister Gowin protested an attempt to go ahead with the elections according to the April 6 bill and resigned from the office. At the same time, it became clearer that conducting elections according to the April 6 bill is technically impossible but some bystanders - especially the election administration - did not want to participate in a political conflict.

A day before the scheduled final vote over the new election law, a new consensus emerged for delaying the elections, and resulted in an agreement between Jarosław Gowin and Jarosław Kaczyński, the leader of PiS. Kaczyński accepted that the elections had to be delayed. Immediately after the agreement had been sealed, the National Electoral Commission announced that the organizational and legal difficulties prevent the elections from happening. On May 7, the Senate’s veto of the electoral law amendment was overridden by the Sejm. The president signed the bill the following day, but it was clear by that time that no time was left for finalizing the election. The plan returned to the initial, traditional election strategy, along with an option that would allow all voters to vote by mail. The National Electoral Commission, which included both members of the opposition and the government, unanimously asked the Sejm’s speaker to set the new election date. All interested parties accepted this solution. The opposition was incentivized to accept the conciliatory solution since they got an option to withdraw their candidate. Instead of the extremely poor-performing Małgorzata Kidawa-Błońska they chose the dynamic president of Warsaw Rafał Trzaskowski. The pressure from the voters, who were increasingly more tired with the conflict, was important as well also (Rychard & Haman 2020). Despite remaining legal uncertainties (Rakowska-Trela 2020), on May 28, after weeks of hot-temperature political drama, all the main candidates supported holding the election on June 28, 2020.

The elections resulted in a close victory of the incumbent, Andrzej Duda, and an impressive appearance of the Platforma’s candidate, Rafał Trzaskowski. The long-term effect was a deepened tension inside the ruling coalition that finally resulted a year later in Gowin’s party, Porozumienie (Agreement), leaving the coalition. As a result, the coalition lost its Sejm majority. In the opposition’s camp, Trzaskowski’s position was strengthened.

The critical veto player turned out to be the local governments, which were dominated by independents and opposition, and which had been organizing the elections for the past 30 years. Government’s attempts to bypass these local governments failed due to the Senate’s prolonged inaction. Although the Senate was unable to block the new electoral law outright, it was able to delay it from proceeding—long enough for it to become irrelevant.

Our conclusion goes against the common opinion that electoral engineering is always one-sided. The reconstruction of the pre-electoral political maneuvers shows that many independent players were simultaneously involved in complex engineering, and that the final outcome was hard to predict until almost the very end.

## Data Availability

Publicly available data were used.

## Footnotes

1 The statistics of COVID-19-related deaths painted a similar picture. Sixty days after passing the threshold of one case per million individuals in a country, the death toll per one million was as follows: Italy—402; France—354; Germany—78; Poland—21; Romania—43; Spain—491; and the UK—432 (WHO 2020).
